# dNTPpoolDB: a manually curated database of experimentally determined dNTP pools and pool changes in biological samples

**DOI:** 10.1093/nar/gkab910

**Published:** 2021-10-13

**Authors:** Rita Pancsa, Erzsébet Fichó, Dániel Molnár, Éva Viola Surányi, Tamás Trombitás, Dóra Füzesi, Hanna Lóczi, Péter Szijjártó, Rita Hirmondó, Judit E Szabó, Judit Tóth

**Affiliations:** Institute of Enzymology, Research Centre for Natural Sciences, Budapest, H-1117, Hungary; Institute of Enzymology, Research Centre for Natural Sciences, Budapest, H-1117, Hungary; Cytocast Kft., Vecsés, Hungary; Institute of Enzymology, Research Centre for Natural Sciences, Budapest, H-1117, Hungary; Institute of Enzymology, Research Centre for Natural Sciences, Budapest, H-1117, Hungary; Institute of Enzymology, Research Centre for Natural Sciences, Budapest, H-1117, Hungary; Institute of Enzymology, Research Centre for Natural Sciences, Budapest, H-1117, Hungary; Institute of Enzymology, Research Centre for Natural Sciences, Budapest, H-1117, Hungary; Institute of Enzymology, Research Centre for Natural Sciences, Budapest, H-1117, Hungary; Institute of Enzymology, Research Centre for Natural Sciences, Budapest, H-1117, Hungary; Institute of Enzymology, Research Centre for Natural Sciences, Budapest, H-1117, Hungary; Institute of Enzymology, Research Centre for Natural Sciences, Budapest, H-1117, Hungary; Department of Applied Biotechnology and Food Sciences, Budapest University of Technology and Economics, Budapest, H-1111, Hungary

## Abstract

Stimulated by the growing interest in the role of dNTP pools in physiological and malignant processes, we established dNTPpoolDB, the database that offers access to quantitative data on dNTP pools from a wide range of species, experimental and developmental conditions (https://dntppool.org/). The database includes measured absolute or relative cellular levels of the four canonical building blocks of DNA and of exotic dNTPs, as well. In addition to the measured quantity, dNTPpoolDB contains ample information on sample source, dNTP quantitation methods and experimental conditions including any treatments and genetic manipulations. Functions such as the advanced search offering multiple choices from custom-built controlled vocabularies in 15 categories in parallel, the pairwise comparison of any chosen pools, and control-treatment correlations provide users with the possibility to quickly recognize and graphically analyse changes in the dNTP pools in function of a chosen parameter. Unbalanced dNTP pools, as well as the balanced accumulation or depletion of all four dNTPs result in genomic instability. Accordingly, key roles of dNTP pool homeostasis have been demonstrated in cancer progression, development, ageing and viral infections among others. dNTPpoolDB is designated to promote research in these fields and fills a longstanding gap in genome metabolism research.

## INTRODUCTION

DNA is a polymer synthesized from four distinct types of deoxyribonucleotide triphosphate (dNTP) monomers. The genetic code is built upon the sequence of these four types of monomers. Predominantly and most often, DNA is constituted of the so-called canonical dATP, dGTP, dCTP and dTTP nucleotides. In addition to that, non-canonical building blocks mimicking one of the canonical ones can be built in by DNA polymerase enzymes. dNTP incorporation rate and fidelity depends largely on the pool of canonical and non-canonical dNTPs available for a given polymerase (1,2). Mutagenesis has been shown to be greatly stimulated either by unbalanced dNTP pools or, intriguingly, by balanced accumulation of all four dNTPs (3–5). On the other hand, proportional depletion of dNTP pools causes genomic instability, probably through replication stress (6). Therefore, the available concentration of DNA building blocks is maintained in homeostasis for proper DNA replication and repair.

The ever-growing interest in cellular dNTP pools can be accounted to the fact that they influence several core biological processes, including the progression of the cell cycle during development (7) and differentiation (8) as well as DNA damage response (9). dNTP pool alterations lead to genomic instability (10) and have been directly linked to ageing (11) and several human diseases, including mitochondrial disorders (12,13), viral infections and immunity (14–17), and cancer (18). Cancer research is a key field benefitting from the accumulated knowledge on dNTP homeostatic changes. The spontaneous mutation frequencies in tumour tissues are at least 200-fold higher than those in normal tissues from which they were derived (19) and this phenomenon is linked to imbalanced dNTP pools. The importance of balanced dNTP pools is also well supported by the fact that some of the key metabolic enzymes controlling them are important targets of cancer therapeutics (17,20–23).

dNTP metabolism has been researched for over 50 years. Even though an astonishing amount of measured dNTP pool data have been accumulated in the literature, they have not been systematically identified and extracted from it until now. The great diversity in the applied methodology, data quality and presentation make it complicated to grab, order and structure these data. Here, we introduce a novel database called dNTPpoolDB. Stimulated by the growing interest in the role of dNTP pool changes in physiological and malignant processes, we constructed this resource on hitherto measured cellular dNTP quantities and pools from a wide range of species, experimental and developmental conditions. The database contains quantitative data on the four canonical building blocks of DNA as well as on exotic dNTPs. dNTPoolDB is manually curated, each entry is extracted from the literature by a competent annotator. When constructing the information components of the database entries, emphasis was given to be able to offer more than just a catalogue. Functions such as the user-friendly advanced search, the pairwise comparison of any chosen pools and control-treatment correlations provide users with the possibility to quickly recognize changes in the dNTP pools in function of a given parameter they are interested in. A keen interest to detect changes in dNTP levels upon stress, treatments or in altered genetic backgrounds is obvious from the number of papers dealing with this. This interest is also indicated by the recent development of several new or modified experimental methods to tackle the challenging determination of the absolute or relative levels of dNTPs in biological samples (24–27).

dNTPpoolDB provides a wealth of ordered and unambiguous information on dNTP measurements in biological samples through a user-friendly web interface that meets the standards of modern biological databases, extended with state-of-the-art features such as cross-link references and mobile responsivity. To the best of our knowledge, no resource exists with similar content and therefore, dNTPpoolDB fills a long-standing gap in the nucleotide and genome metabolism research fields. dNTPoolDB is designated to promote research in the fields of DNA replication and repair, cancer research, development and ageing, viral and bacterial infections and in the yet undiscovered aspects of dNTP pool homeostasis, comprising the potential signalling function of dNTPs.

## DATA COLLECTION AND DATABASE CONTENT

To find relevant data, a large number of publications with the keywords matching several expressions for dNTPs in the title and abstract was initially retrieved from the PubMed database (28). Then we manually filtered this article pool to obtain the pertinent original publications containing experimentally determined dNTP values. We reviewed the full text of the publications to localize measured dNTP data in tables, figures, and in-text descriptions. Our objective was to retrieve as much information as possible related to the measured dNTP values in a structured way. Whenever possible, we used ontologies and custom-built controlled vocabularies. All information taken from the literature through manual curation is linked to corresponding manuscripts via PubMed (28). Each entry represents a single measured dNTP value.

**Figure 1.**
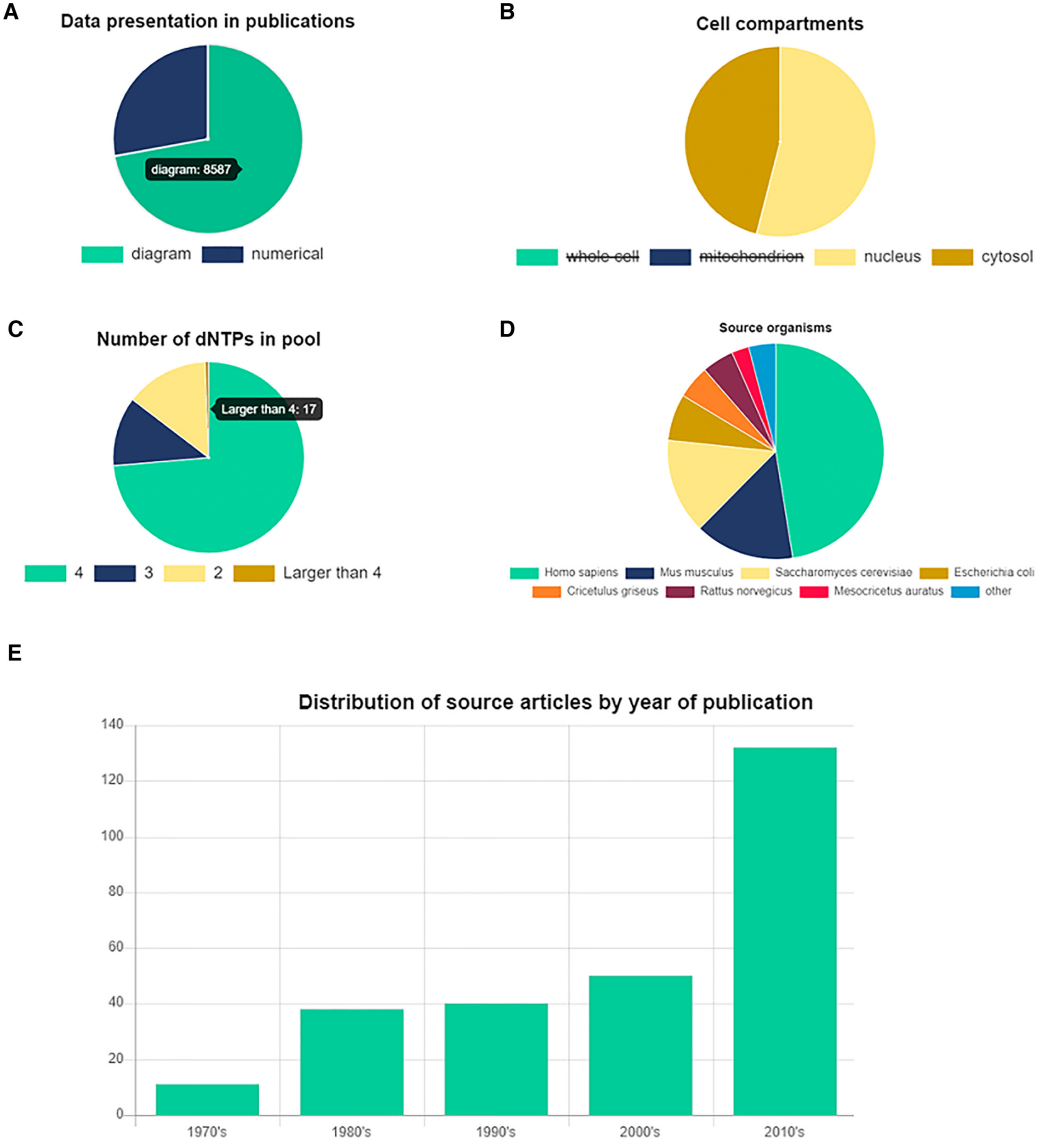
Statistics and customizable statistical analysis of the collected data. (**A**) Distribution of the graphical and numerical representations of the data in the source literature. By scrolling over a pie, the number represented by the pie shows up. (**B**) Using the customizable pie charts: deselecting can be done by clicking on the legend. (**C**) Distribution of the number of dNTPs in the pool. (**D**) Source organisms represented in the database. (**E**) Time distribution of the number of papers annotated so far.

During data collection, it was a challenge to extract the measured values and their errors from figure diagrams. As shown in Figure 1A, about 70% of the data was graphically presented in the literature. To retrieve graphically presented data, we used WebPlotDigitizer 4.4, a free online tool developed by Ankit Rohatgi (https://automeris.io/WebPlotDigitizer). By converting several thousands of graphical dNTP quantitation points into numerical format, we made these valuable data accessible for subsequent bioinformatic analysis. Graphical to numerical representation conversion is indicated in the entry pages.

Another challenge was the management of dimensions due to the large variety of sample sources, measurement methods and data quality. To increase data comparability within the database, we reduced the number of dimensions used without transforming the data in a subjective way. We only allowed order of magnitude conversion, the conversion of fractional representation to percentage, and the conversion of expressions of relative changes into fold change. We created an ‘other dimension’ category for rarely occurring dimensions. dNTP quantification data are often presented in the literature as relative values compared to a non-treated control or to other nucleotides (ATP, dNTP or NTP content typically). In these cases, we indicated the basis for the comparison. We found it particularly important to identify control-treatment pairs in case of the relative data to increase the information content of the database.

Error values were also extracted from the papers, where available. Error values and error types are also given in the database to provide information on the reliability of the presented values.

We intended to unambiguously describe the source of the measured biological sample at different levels from organism to subcellular compartment. The Taxonomy Browser ID of NCBI is cross-referenced for each organism. Where possible, we included as a cross-reference the Cell Line Ontology (CLO) number with links to the corresponding EMBL-EBI Ontology Search (OLS) entry.

As method developers ourselves, we found it important to identify and indicate the conditions for sample extraction and the measurement methods. dNTP measurement methods all have their biases and limitations and therefore, the included methodological information may come useful when considering the robustness of the data, or when comparing data.

dNTPpoolDB also describes relevant experimental conditions. Most studies inquire about dNTP pool changes emerging as a result of some treatment or naturally occurring genetic or environmental alterations. Therefore, we indicate the fact of treatment and its nature. If it is a drug treatment, we identify the drug using cross-reference to its PubChem ID (29). If the treatment is genetic manipulation, we identify the affected genes and proteins using cross-references to NCBI’s Gene IDs and to the UniProt IDs, respectively, together with information on their mutations. When more than one gene is affected, or more than one type of genetic manipulation was done in a sample, these are shown in subsequent sections (e.g. consult entry DNTP000512). Other details of the applied treatments are provided as free text. Within the free text, we intended to yield information about the pathways or processes affected by the drug treatment or genetic manipulation (e.g. thymidylate synthesis, dNTP biosynthesis, oxidative stress). These free text additions are searchable.

**Figure 2.**
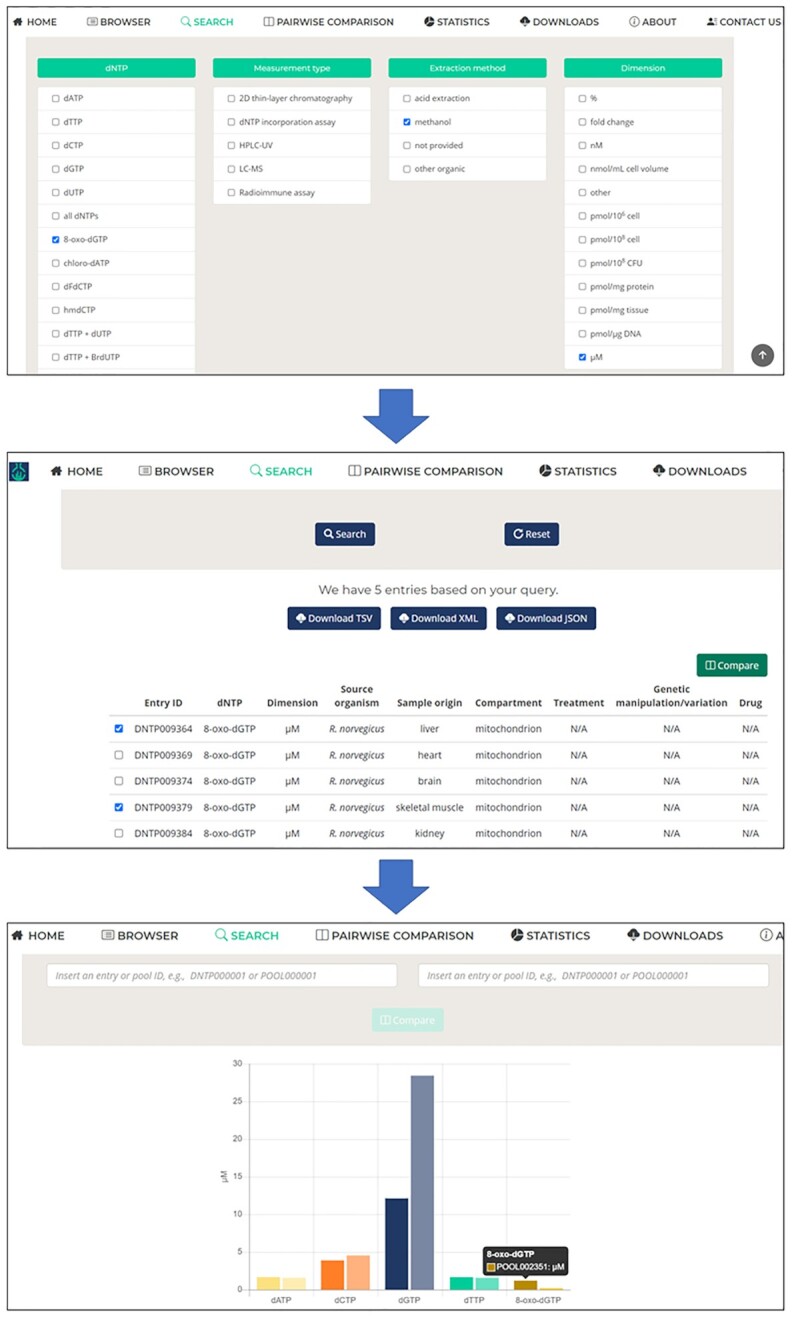
Featured functions of the user interface of dNTPpoolDB. The Advanced search function offers multiple choices of a predefined set of parameters to narrow down data selection in response to a precise query. Upon selecting any two of the search results from the list, one can compare the dNTP pools that belong to the two selected entries by hitting the Compare button. Then, the compared data will graphically appear in a new window. If dimensions match, data will be presented in a single chart. If dimensions are different, two charts will be shown with an alert not to compare these data directly. Below the chart, all the information contained by the two compared entry pages will be displayed side-by-side.

dNTP pools are reconstructed from single entries. Those entries that differ only in the type of measured dNTP belong to the same pool. Each dNTP pool has a pool ID. Pools are further related to each other by the definition of control-treatment pairs, where applicable. In addition to the possibility to compare and graphically represent control-treatment pairs, any two pools can be compared, as well (Figure 2).

## USER INTERFACE

### Overview of functions

dNTPpoolDB provides a simple and intuitive interface to browse, search, visualize, compare, and download detailed information about dNTP measurements (Figure 2). Care was taken to use the same colour code for consistency throughout the pages. The tool bar on the Home page offers access to each of the functions of the database. The Browser page is organized as an interactive and sortable table that allows users to quickly browse through the entire or a subset of the entries. Column headers contain options that help to quickly make a complex selection of entries. Clicking on any row inside the Browser table directs the user to the relevant entry page.

The Advanced search page can be reached from the Browser page or from the constantly available tool bar. It allows free text search using multiple keywords as well as search by structured, predefined parameters. These parameters are organised into tabs that contain a drop-down menu (Figure 2). The user can make multiple choices in each of the drop-down menus thus making a complex selection possible (Figure 2). The result of the selection can be downloaded from here.

The pairwise comparison function is useful for quickly assessing differences between two entries and to detect dNTP pool changes in function of a desired parameter. This function can be reached (i) through the Browser page, (ii) through the Advanced search page, (iii) from the Entry page and (iv) directly in the dedicated Pairwise comparison page. In the Browser and Advanced search pages, one can select two entries to be compared using the selection box on the left (Figure 2). Then by hitting the Compare button on the upper right, the Pairwise comparison page shows up with the result. Although individual entries are selected, the entire pools are compared if applicable (Figure 2). If the dimensions of the measured dNTP values match, the comparison is done in a single bar chart (Figure 2). We alert users whenever datasets are not directly comparable. Below the graphical comparison, the user finds all information available in the Entry pages in a transparent comparison layout. A useful workflow is shown in Figure 2, whereby the user performs a narrow selection of entries using the predefined parameters of the Advanced search function, then chooses two entries from the results list and compare them. Alternatively, the user has the option to introduce two entry or pool identifiers into the relevant query boxes in the Pairwise comparison page and hit the compare button. The discussion of using the compare function from the Entry pages brings us to control-treatment pairing. Whenever we could identify control and treatment measurements in the source publication, we aimed to pair these data. We believe that providing these relationships between different entries will largely facilitate all future analyses of the collected data. When control-treatment pairs are available, the compare button is offered directly below the related pool in the Entry pages to make the graphical comparison of the cohesive pools effortlessly feasible.

The Statistics page provides useful summaries of dNTPpoolDB data from different aspects to provide a quick overview and to facilitate the planning of future data analyses by users, relying on the entirety or any subset of the available data. The pie charts of the Statistics page are customizable. The user may choose the option to visualize a subset of the categories in the pie charts (Figure 1B). Selecting and deselecting a category can be done by clicking on the legend. For example, if the user is interested in visualizing the distribution of the number of dNTP measurements between the cytosol and the nucleus, it can be done by deselecting the other two categories, i.e., the whole cell and the mitochondrion (Figure 1B). That way, the information practically lost in the entire set of these four statistical categories due to the dominance of the large number of entries originating from whole cells can be retrieved. Also, by scrolling over a pie in the pie chart, the user can visualize the number represented by that specific pie (Figure 1A). dNTPpoolDB allows users to download all the obtained data in the Download page in three different formats: TSV (tab-separated version), standard XML and JSON format. In addition, single data points can be downloaded directly from the Entry pages and a selected subset of data from the Advanced search page. A comprehensive online documentation about the use and functionalities of dNTPpoolDB is available in the About page. To encourage users to help in complementing the database, dNTPpoolDB offers a Contact page that enables researchers to submit publication IDs and other information pertinent to the dNTP data content of the proposed source literature.

### Entry pages

Each entry in dNTPpoolDB corresponds to a single dNTP measurement with a dedicated entry page containing all relevant information collected manually from the literature. The header of the entry page displays the ID of the entry and the type of dNTP measured. Within the same header, one can also jump to the previous or the following entries by clicking on the displayed IDs. The top right green bar provides the option of downloading the annotation of the given entry in JSON, TSV and XML formats. On the left, the chemical structure of the measured dNTP(s) can be seen (retrieved from PubChem (29)).

The Results section of the entry page contains the actual measured value, its error and dimension. If the value is a relative one, the basis of comparison is indicated in the ‘Relative compared to’ row. Whether the data point was presented numerically or graphically is also indicated here. This section contains two cross-references to PubMed and PMCID to reach the source article in a new browser page.

The Source section serves to identify the origin of the biological sample in which the measurement was done. Here, we provide a link to the taxonomic identification of the organism and specify further at subsequent organisational levels the origin of the biological sample. In each case, we use the name of the bacterial, fungal and animal strains, that of the cell lines or primary cells, tissues as given in the source article. In addition, if it is possible, we provide the CLO link for cell lines, e.g. Tango cell (CLO:0037271), for HEK 293T.

The Experimental details section indicates the major type of dNTP quantitation. We defined five main methods comprising the enzymatic dNTP incorporation assay, HPLC–UV, LC–MS, radioimmune assay, and thin layer chromatography. Here, we also indicate the method of dNTP extraction from the biological sample. Any alterations from the predefined methods are indicated in the Remarks to measurement type row. The most often used subtypes of dNTP measuring methods are also referred to in this section (e.g. the method of Sherman and Fyfe is commonly used for dNTP incorporation-based measurements (30).

In the Treatment section, we classified treatments into drug and/or stress treatments and genetic manipulation. Some stress treatments are described in free words as standardization was not always an option. However, in case a chemical was applied on the source organism, we unambiguously specified it using its PubChem link in addition to indicating the drug name as used in the publication. In case the measurement was done following a genetic modification, we indicated the details of this in the Genes and Proteins section. Here, the type of genetic modification/variation (e.g. silencing, overexpression, mutation) and all reported affected genes and proteins are identified using Gene and UniProt IDs, respectively, in addition to indicating the gene and protein names given in the article. If mutated forms of the proteins were applied in the measurement, those are also described. The Treatment details/effects comment potentially also contains information on the effects of the applied mutations or drugs. This and other comments section may be useful when users search the database using the free word finder.

Entries are grouped into identifiable pools whenever the levels of at least two different dNTPs have been measured in the same study under the same conditions. Where available, pool information is also included into the entry pages in the dNTP Pool section on the left-hand side. Here, all linked measurements can be reached via a link to the relevant entry page. The reconstituted pool is graphically represented in a colour coded bar chart. Scrolling over the bars will show the represented value with its dimension. If the presented data is part of a control-treatment pair within the database, then the corresponding control or treatment pool will also be shown in the panel right below the linked pool members. In this case, the compare button will also show up. By hitting the Compare button, control-treatment pairs will be automatically compared.

## DATABASE STATISTICS

The current version of dNTPpoolDB contains 11895 individual entries incorporated into 2968 pools, annotated from 283 publications. 78% of the pools contain the four canonical dNTPs (Figure 1C). The number of dNTPs in a pool may be larger, typically five if dUTP is also measured. Exotic dNTPs are often measured separately. It is not rare that authors are interested in measuring only two dNTPs (Figure 1C).

In some cases, the number of data represented by the pie charts are greater than the total number of entries in the database. This only means that non-exclusive categories are involved and thus one entry may fall into more than one category.

More than half of the data comes from human samples, followed in abundance by mice, yeast and *E. coli* samples (Figure 1D). We counted the number of publications that contain dNTP measurements each year from 1970 to 2020 and found that the number of dNTP-related studies steadily grow until the 2010’s, when a burst is observed (Figure 1E).

## SYSTEM DESIGN AND IMPLEMENTATION

dNTPpoolDB can be accessed through a user-friendly, appealing DJANGO (version 3.2.6) based web interface, backed by a highly efficient multi-layer SQL database. dNTPpoolDB is compatible with the various devices and browsing options users usually use (the front-end is implemented in a combination of Bootstrap (version 3.1.0) and JQuery (version 3.5.1)). Besides providing access to the data through the online interface, those can also be downloaded in JSON, XML or TSV formats.

## CONCLUSIONS

dNTPpoolDB is a unique resource with its purpose and content. Our effort to establish dNTPpoolDB was prompted by the need of researchers to access a comprehensive dataset on dNTP quantitation accumulating for a long time. We believe that it fills a long-standing gap in the nucleotide and genome metabolism research fields and will hopefully help answering basic questions related to the diverse roles of dNTP homeostasis. Having overcome the technical difficulties imposed by the diversity in quality, methodology and presentation of published dNTP measurements, we created a reliable manually curated resource. dNTPpoolDB is intended to benefit the scientific community by providing (i) a freely accessible, easy-to-use, efficiently organized resource with relevant data on published cellular dNTP levels and pools, (ii) a good basis for objective comparisons between and assessment of widely applied dNTP measurement methodologies, potentially leading to improved standardization, (iii) ample data for identifying and understanding the species-specific features of dNTP pools, their changes upon different treatments and their role in the development of pathological conditions, 4) clues to the potential non-canonical functions of dNTPs, e.g. in cell signalling and epigenetics.

## DATA AVAILABILITY

dNTPpoolDB is an open resource database available at https://dntppool.org/.

Our aim is to maintain a regularly updated online resource by continuously incorporating published dNTP data. To successfully accomplish this goal, we kindly encourage the scientific community to draw our attention to not yet annotated data via the Contact form (https://dntppool.org/contact/). Data collection from users and submitters through the server is executed via a secure interface using HTTPS, and fully adheres to the General Data Protection Regulation (GDPR) of the EU.

The article refers to the following online tools and resources:

https://automeris.io/WebPlotDigitizer

https://pubmed.ncbi.nlm.nih.gov/

https://europepmc.org/

https://www.ncbi.nlm.nih.gov/taxonomy

https://pubchem.ncbi.nlm.nih.gov/

https://www.ncbi.nlm.nih.gov/gene/

https://www.uniprot.org/

https://www.ebi.ac.uk/ols/index
